# A Complete Occlusion of Right Coronary Artery Due to Stanford Type A Aortic Dissection - Successful Treatment with Extracorporeal Membrane Oxygenation (ECMO)

**DOI:** 10.21470/1678-9741-2018-0060

**Published:** 2019

**Authors:** Yong Wang, Zhicheng Zhu, Rihao Xu, Dan Li, Tiance Wang, Kexiang Liu

**Affiliations:** 1 Second Hospital of Jilin University, Changchun, China.

**Keywords:** Stanford Type A Aortic Dissection, Coronary Involvement, Extracorporeal Membrane Oxygenation (ECMO)

## Abstract

We present a patient diagnosed Stanford Type A aortic dissection, who was misdiagnosed as acute myocardial infarction for 5 days. In the surgery, the right coronary ostium was totally occluded, and the right coronary artery (RCA) was bluish from the trunk to branches. The true lumen couldn’t be found when we opened the RCA. We had to give up coronary artery bypass grafting (CABG). After a regular surgery of type A aortic dissection, the patient was failed to wean from cardiopulmonary bypass due to the right heart dysfunction. The Extracorporeal membrane oxygenation (ECMO) was instituted. The right ventricular wall motion was gradually improved during the post-operation period. This is the first report of using ECMO to successfully treat a complete occlusion of the right coronary artery due to a Type A aortic dissection. This case demonstrates the value of ECMO in assisting right heart function to ensure stable hemodynamics and myocardial recovery in the type A aortic dissection with coronary involvement.

**Table:** 

Abbreviations, acronyms & symbols	
BP	= Blood pressure
CABG	= Coronary artery bypass grafting
CPB	= Cardiopulmonary bypass
CTA	= Computerized tomography angiography
CVP	= Central venous pressure
ECG	= Electrocardiogram
ECMO	= Extracorporeal membrane oxygenation
ICU	= Intensive Care Unit
RCA	= Right coronary artery
TTE	= Transthoracic echocardiogram

## INTRODUCTION

Stanford type A aortic dissection is a life-threatening emergency, which usually presents with acute onset of sharp chest pain. The incidence of aortic dissection is 3.5/100,000^[1]^. The most common risk factor is hypertension. Other risk factors include preexisting aortic diseases or aortic valve disease, family history of aortic diseases, history of cardiac surgery, cigarette smoking, direct blunt chest trauma and use of intravenous drugs^[2]^. Rapid diagnosis and treatment of acute type A aortic dissections is critical. Dissections involving the coronary arteries result in higher risk of death and frequently present with nontraditional symptoms. We present a rare case of a 36-year-old woman with a complete occlusion of right coronary artery (RCA) due to a Type A aortic dissection.

## CASE REPORT

**Fig. 1:**
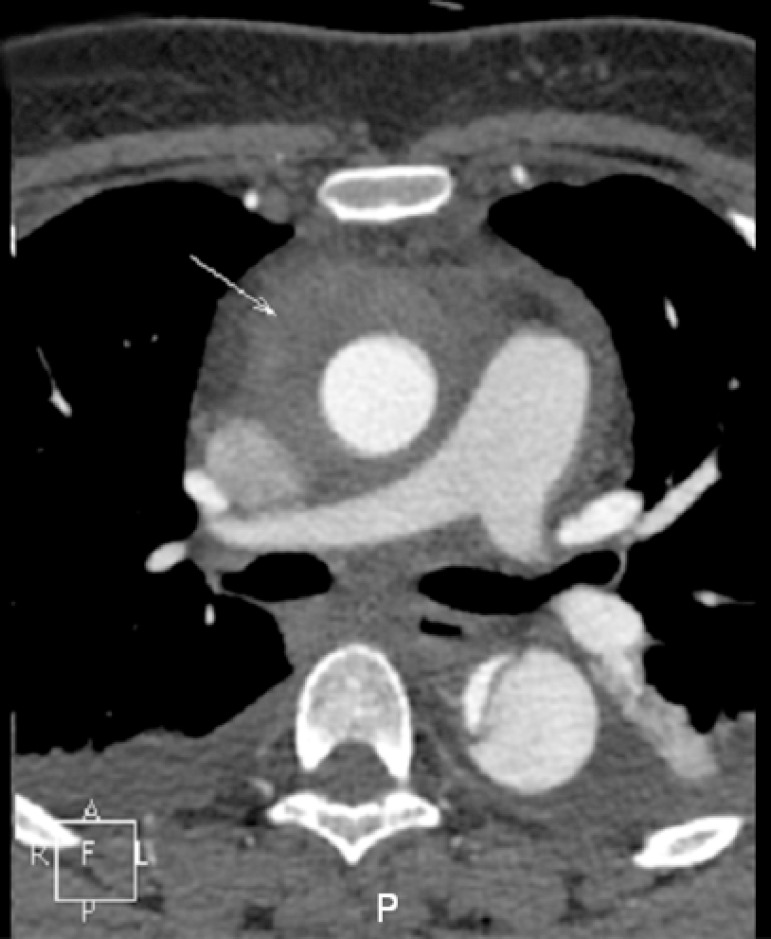
Preoperative Computerized tomography angiography showing ascending and descending aortic dissection.

A 36-year-old woman was admitted to a township hospital following a sharp chest pain and profuse sweating. Her medical history included longstanding arterial hypertension. Twelve-lead electrocardiogram revealed an ST-segment elevation in the II, III and aVF leads. The diagnosis of acute inferior myocardial infarction was considered, and the patient was immediately transferred to a local county hospital. A cardio-pulmonary resuscitation was performed in an ambulance for the sudden onset of cardiac arrest during the transfer. The vital signs recovered and the hemodynamic was stable. On day 6, a magnetic resonance imaging was conducted for the persisting and uncontrolled chest pain and she was diagnosed having a type A aortic dissection. She was transferred to our hospital by an ambulance. On arrival, her heart rate was 110 beats per minute and blood pressure (BP) was 80/45 mmHg. The Computerized tomography angiography (CTA) confirmed a Stanford Type A aortic dissection (Figure 1) and moderate pericardial effusion was also identified. The dissecting space of the aorta was thrombotic from the aortic root to the aortic arch, and the dissection extended from the arch to the level of the iliac artery. The electrocardiogram showed a pathologic Q wave in the III and aVF leads. Therefore, the emergent surgery was arranged for this patient immediately.

**Fig. 2:**
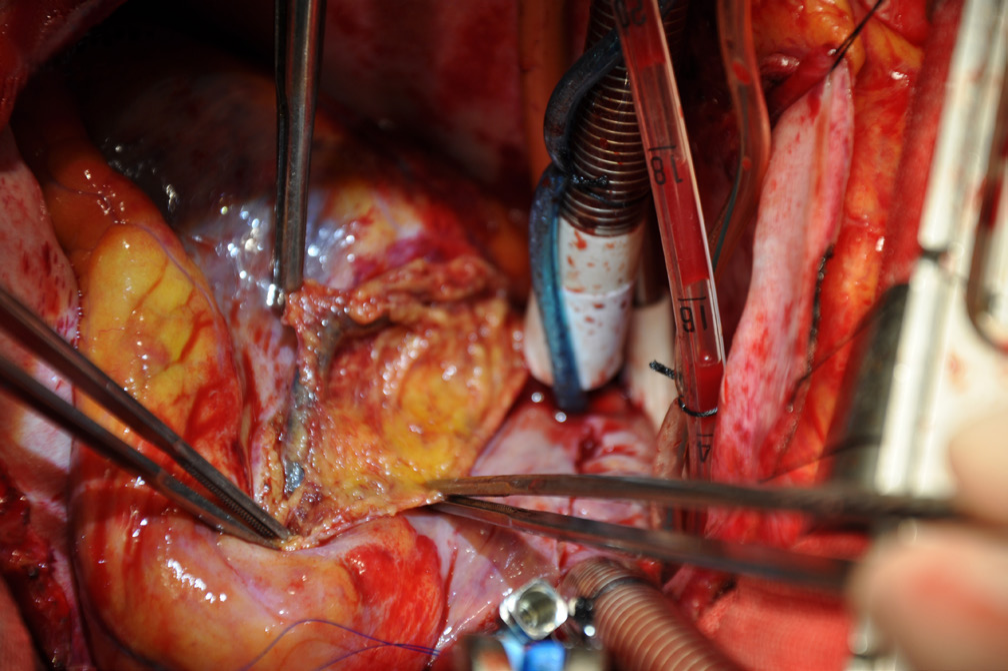
An intraoperative photograph demonstrating that a complete thrombosis of the right coronary artery turned bluish.

The surgery was performed in median sternotomy. After pericardiotomy, about 150 ml of bloody pericardial effusion was removed. The surface of the whole heart and the dilated ascending aorta were bluish. The right ventricle was motionless. Once a cardiopulmonary bypass (CPB) was established, systemic cooling was initiated. The ascending aorta was clamped just proximal to the brachiocephalic artery and transected above the sinotubular junction. When beginning to perfuse the heart with myocardial protection solution directly through the coronary ostium, we found that the right coronary ostium was totally occluded. Therefore, we opened the right atrium and administered cold blood cardioplegic solution retrogradely through the coronary sinus and antegradely through the left coronary ostium. The heart was arrested completely. After dissecting the RCA, we found RCA was bluish from the trunk to branches. The true lumen couldn’t be found when we opened the RCA (Figure 2). We had to give up coronary artery bypass grafting (CABG). When rectal temperature decreased to 28℃，the circulation was arrested. The primary entry tear was found in the lesser curvature of aortic arch. The selective cerebral perfusion was performed through right axillary artery cannulation and left common carotid artery. A self-designed 24 mm self-expandable elephant stent graft was inserted into the true lumen of the descending aorta as the elephant trunk, and the free vascular graft was located inside the native arch. The portion of vascular graft, which covered the orifices of 3 vessels, was wedge-shaped resected. A continuous suture (5-0 Polypropylene) was performed inside the native arch from the lower edge of the left subclavian artery to the two sides and ran through the entire native vessel wall around the orifices of the 3 branches to attach the vascular graft to the native arch. The anastomosis of the proximal of aortic arch and the vascular graft using a 24-mm Dacron graft with an 8-mm side branch was accomplished with a continuous suture. Then the prosthesis was de-aired, we restarted a full CPB through the side branch of the graft, and the patient was gradually rewarmed. The anastomosis of the reinforced ascending aortic stump and the vascular graft was accomplished with a continuous end-end suture. Blood pressure and heartbeat of the patient dramatically dropped during the removal of CPB due to the large area of myocardial infarction in the right heart, although maximal doses of vasopressor and inotropic support (dopamine 20.0µg/kg/minute, epinephrine 0.3µg/kg/minute) were given. The patient was failed to wean from CPB. Peripheral VA-ECMO was implanted with a 22-French Femoral Venous Cannula (Edwards Lifesciences, Irvine, CA, USA) in the right femoral vein and a 24-French Arterial Perfusion Cannula (Edwards Lifesciences) grafted to the right femoral artery via an 8 mm beveled Gelweave™ graft (Terumo, Scotland, UK)". The ECMO circuit consisted of a centrifugal pump (Revolution 5; Sorin Group, Mirandola, Italy) with a membrane oxygenator (HLS module advanced; Maquet, Hirrlingen,Germany). When the ECMO (Pump Rotation: 2367R/min;Blood Flow: 3.880 L/min) started, we stopped the routine CPB. The patient BP was 89/49 mmHg and central venous pressure (CVP) was 5cm H_2_O with inotropic support (dopamine 20.0µg/kg/minute, epinephrine 0.3µg/kg/minute, nitroglycerin 2.0µg/kg/minute). The chest was closed in the usual fashion. The time of selective cerebral perfusion was 44 minutes, the total circulatory arrest time was 45 minutes, and the CPB time was 239 minutes. When the patient was taken to the ICU, the BP was 90/70mmHg and CVP was 11cm H_2_O. Three hours later, the patient recovered consciousness. The patient was extubated in 18 hours post-operation. We regularly monitored the changes of cardiac function through bedside transthoracic echocardiogram (TTE). The movement of the right ventricular wall gradually improved. On day 13 post-operative, ECMO was removed. The final postoperative TTE revealed that the left ventricular ejection fraction was 55% and the right ventricular wall moved normally. The patient recovered uneventfully.

**Fig. 3:**
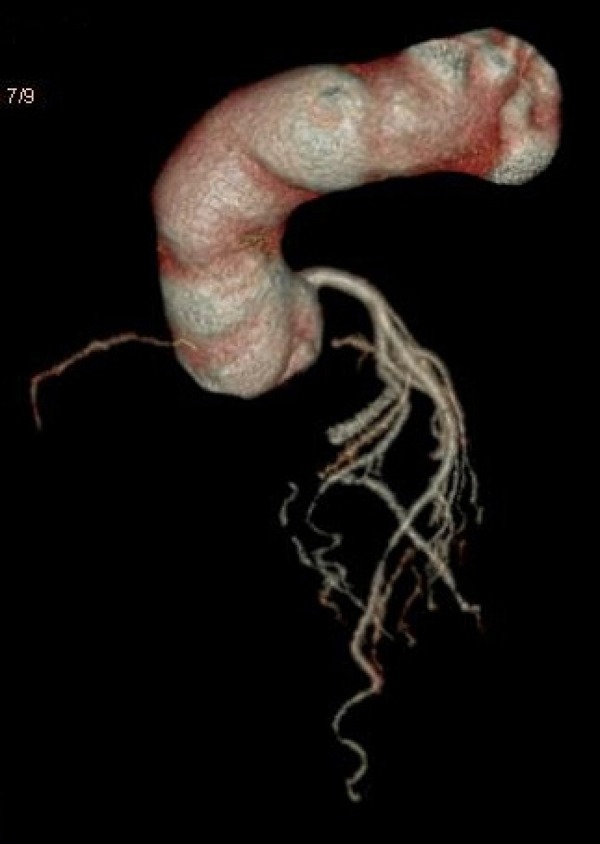
A follow-up CTA demonstrating that the right coronary artery was undeveloped and only a short period of residual cavity remained.

The patient had scheduled a follow-up three months after discharge. The CTA demonstrated that the RCA was totally occluded, except a small caliber true lumen visualized (Figure 3).

## DISCUSSION

A coronary involvement in type A aortic dissection is a serious phenomenon in clinic, which presents in 10-15% patients with aortic dissection^[2]^. Clinical research has shown that the mortality rate of a type A aortic dissection involving the coronary artery is high (up to 20% ~ 33.3%) after surgery^[3,4]^. According to Neri et al.^[5]^, there are three mechanisms of total occlusion of right coronary ostium: type A, where ostial dissection is defined as a disruption of the inner layer limited to the area of the coronary ostium; type B, a dissection extending into the coronary artery; and type C, a coronary disruption (intimal detachment). The ideal treatment for this condition is surgery with ascending aorta replacement and CABG or a coronary repair, if possible. In our patient, acute Type A aortic dissection was accompanied with myocardial infarction and pericardial effusion. Without prompt treatment, it will result in death due to myocardial infarction or aortic rupture. The RCA dissection was already suspected based on the presence of pathologic Q on ECG on the patient’s arrival. But in the surgery, we found RCA was bluish from the trunk to branches, and the RCA was totally thrombosed. The true lumen couldn’t be found when we opened the RCA. There was no place available for CABG on the RCA. The follow-up CTA also demonstrated that the RCA was totally occluded, except for a small caliber true lumen visualized. After a regular surgery of Stanford type A aortic dissection, the patient was difficult to wean from CPB due to the right heart dysfunction. In this case, we excluded consideration of an intra-aortic balloon pump because of the residual dissected thoracoabdominal aorta. The only option left was to continue to use assisted circulation which helped myocardial recovery. The right ventricular circulatory support was another option, but was not approved for use in China. Besides, we had more experience on installing ECMO on patients with right ventricular failure and got satisfied outcomes. So, we chose ECMO to assist right heart function. As expected, the hemodynamic parameters were favorable after initiation of ECMO, and the right ventricular wall motion was gradually improved during the post-operation period.

This is the first report of using ECMO to successfully treat a complete occlusion of the RCA, when CABG can’t be performed due to a Stanford Type A aortic dissection. This case demonstrates the value of ECMO in assisting right heart function to ensure stable hemodynamics and myocardial recovery in the type A aortic dissection with coronary involvement.

**Table:** 

Author's roles & responsibilities	
YW	Collected the date and wrote the article; final approval of the version to be published
ZZ	Referred to the related literature; final approval of the version to be published
RX	Referred to the related literature; final approval of the version to be published
DL	Referred to the related literature; final approval of the version to be published
TW	Referred to the related literature; final approval of the version to be published
KL	Designed the study; performed the operation; and revised the manuscript; final approval of the version to be published
